# Dual-wavelength pump-probe microscopy analysis of melanin composition

**DOI:** 10.1038/srep36871

**Published:** 2016-11-11

**Authors:** Andrew Thompson, Francisco E. Robles, Jesse W. Wilson, Sanghamitra Deb, Robert Calderbank, Warren S. Warren

**Affiliations:** 1 Department of Electrical and Computer Engineering, Duke University, Durham, North Carolina 27708, USA; 2 Department of Chemistry, Duke University, Durham, North Carolina 27708, USA; 3 Department of Physics, Duke University, Durham, North Carolina 27708, USA

## Abstract

Pump-probe microscopy is an emerging technique that provides detailed chemical information of absorbers with sub-micrometer spatial resolution. Recent work has shown that the pump-probe signals from melanin in human skin cancers correlate well with clinical concern, but it has been difficult to infer the molecular origins of these differences. Here we develop a mathematical framework to describe the pump-probe dynamics of melanin in human pigmented tissue samples, which treats the ensemble of individual chromophores that make up melanin as Gaussian absorbers with bandwidth related via Frenkel excitons. Thus, observed signals result from an interplay between the spectral bandwidths of the individual underlying chromophores and spectral proximity of the pump and probe wavelengths. The model is tested using a dual-wavelength pump-probe approach and a novel signal processing method based on gnomonic projections. Results show signals can be described by a single linear transition path with different rates of progress for different individual pump-probe wavelength pairs. Moreover, the combined dual-wavelength data shows a nonlinear transition that supports our mathematical framework and the excitonic model to describe the optical properties of melanin. The novel gnomonic projection analysis can also be an attractive generic tool for analyzing mixing paths in biomolecular and analytical chemistry.

Melanin is a complex biopolymer with a number of interesting physical and chemical properties that has gathered much interest from both the biomedical and materials science communities^1^. Though it has been extensively studied, the precise details of its chemical structure and biological functions still remain elusive^2^,^3^. This is partly because melanin is difficult to characterize and the gold standard approach for melanin analysis, oxidative degradation followed by mass spectroscopy^4^, does not preserve the spatial arrangement of its chemically distinct constituents over microscopic scales. To overcome some of these limitations, femtosecond transient absorption and nonlinear optical microscopy techniques were combined^5^. This novel approach, called pump-probe microscopy, provides contrast between different types of melanins based on excited state ultrafast photodynamics, with subcellular spatial resolution^6^; specifically, pump-probe microscopy is sensitive to the pigment’s ion content, aggregate size, oxidative stress, and type (eu-/pheo-melanin)^7^. This method has found use in analyzing pigmented lesions for melanoma detection and staging in excised human biopsy specimens^8^,^9^,^10^,^11^ and animal models *in vivo*^12^,^13^. In this work we expand upon this method by analyzing the transient absorption response of melanin with multiple pump-probe wavelengths to (1) determine if the additional information can lead to more specific identification of different types of melanins naturally produced by humans and (2) gain additional insight into their underlying structure.

Pump-probe microscopy uses two ultrafast laser pulses (pump and probe) to interrogate the transient excited-state and ground-state photodynamics of pigmented molecules. In this technique, first the pump pulse electronically excites the molecules and partially depletes the ground state, and then the probe beam monitors the altered molecular states after an allotted time for the electronic population to evolve (in the femto- to pico-second time scales). Figures 1 and 2(a) shows a schematic representation of various pump-probe signals. Our work to date^7^,^14^,^15^ has lead to a hypothetical physical model for the resulting dynamics in melanin that treats the interactions as a spectral hole burning phenomenon in competition with excited state absorption. This model builds upon the idea that the broad absorption profile of melanin comes from a sum of an ensemble of underlying chromophores with overlapping absorption bands^16^. Similar to a spectral hole burning experiment, the pump-probe response is dominated by ground state bleaching (negative signals) when the probe wavelength is *within* the bandwidth of the chromophores that were selectively excited by the pump. Excited state absorption (positive signals), on the other hand, can occur when the probe energy is resonant with a transition from the excited state of the pump-selected chromophore to a higher-lying excited state. Thus, differences in the pump-probe response are ultimately accounted for by differences in the spectral bandwidths of the underlying chromophores excited by the pump, proximity of pump and probe wavelengths, and diversity of neighboring chromophores (see Fig. 1). Recent work by Chen *et al*.^17^ proposed that the absorption spectra of the underlying chromophores in melanin can be described in terms of Frenkel excitonic couplings between the protomolecule building blocks that aggregate to form melanin^17^. Frenkel excitons (as opposed to other types of excitons) are characteristic of molecular aggregates^18^,^19^,^20^. This leads to a mathematical framework that can be directly applied to an analysis of pump-probe data acquired with multiple pump-probe wavelength pairs. Using a robust principal component analysis^21^ and a gnomonic projection to linearize the visualization of the responses^22^, we further verify this excitonic model and show that the transient absorptive behavior of melanin for a given pump-probe wavelength pair can be described by a single linear transition path with different rates of progress for different wavelength pairs. Our model also predicts that when multiple pump-probe wavelength pairs are combined these differences in the rates of progress produce a nonlinear transition–this behavior is verified experimentally.

## Results

### Physical model of melanin pump-probe response

**Figure 1:**
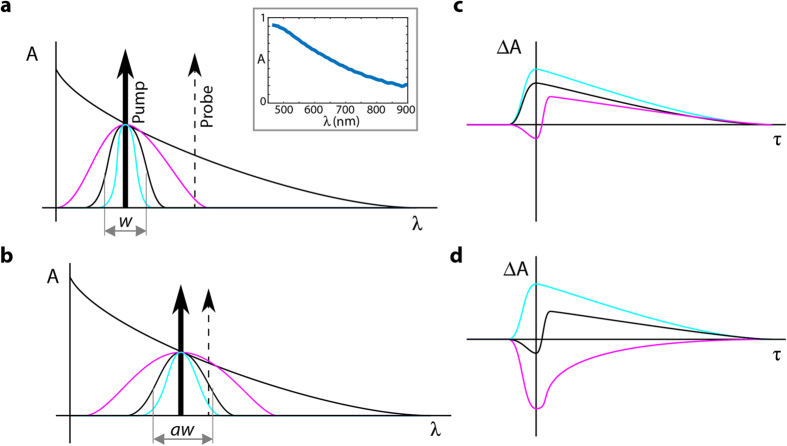
Schematic representation of melanin linear absorption and nonlinear excited-state transient absorption. (**a**,**b)** Representation of melanin linear absorption along with one underlying Gaussian chromophore with three different possible bandwidths. Inset in (**a**) shows an average of experimentally measured linear (i.e., stationary) absorption spectra of melanin in human tissue. (**c**,**d)** Corresponding pump-probe signals using different pump-probe wavelength combinations. (Experimental pump-probe measurement are shown in Figs 3 and 5).

In our model, the pump-probe ultrafast transient behavior results from differences in the spectral bandwidths (and diversity) of the underlying ensemble of chromophores that make up melanin, as well as the spectral proximity of the pump and probe (see Fig. 1). As such, the pump-probe response is dominated by one type of signal (ground-state bleaching, GSB) when the probe is tuned close to the pump wavelength, and dominated by a different type of signal (excited-state absorption, ESA) when the probe is tuned farther from the pump wavelength^7^. Note that ground-state bleaching produces an increase in the transmitted probe intensity, whereas excited state absorption reduces the intensity (i.e., loss); further, pump-probe convention is that lossy signals are positive. As a function of pump-probe time delay τ, the pump-probe response of melanins can be described in terms of two endmembers that happen to possess opposite signs, S_GSB_(λ_pu_, τ) ≤ 0 and S_ESA_(λ_pu_, τ) ≥ 0, each in general a multiexponential function convolved with the cross-correlation of the pump and probe amplitude envelopes. The lifetimes of the excited states are set by the chromophore population that was selected by the pump, thus in our physical model the endmembers are a function of the pump wavelength, λ_pu_, and independent of the probe wavelength, λ_pr_. The overall pump-probe signal’s dependence on λ_pr_ can be explicitly accounted for by its spectral proximity to λ_pu_ relative to the bandwidth of the chromophore excited by the pump. Thus, the pump-probe response can be expressed as,


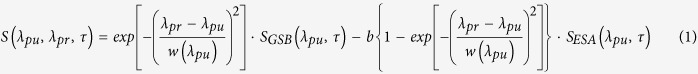


where *w*(λ_pu_) is the bandwidth of the chromophore excited by the pump, modeled as a Gaussian (see Fig. 1), and *b* is related to the relative oscillator strengths of the ground state compared to the excited state transitions. We use a Gaussian model as this represents the spectral hole burned by the pump. Further, because our pump wavelengths presumably hit the near-IR tail of the exciton-broadened chromophores, we avoid most of the complex structures present at lower wavelengths, leaving the spectral hole at λ_pr_ > λ_pu_ approximately Gaussian^23^. (This assumption may not be valid for λ_pr_ < λ_pu_). Note that as the probe wavelength approaches the pump S(λ_pu_, λ_pr_ → λ_pu_, τ) ~ S_GSB_(λ_pu_, τ), and as it is pulled farther away S(λ_pu_, λ_pr_ >> λ_pu_, τ) ~ S_ESA_(λ_pu_, τ), which matches our previous observations^7^,^14^.

### Incorporation of exciton model

For the experiments described below (see methods section), we acquire data with two different pump wavelengths, λ_pu1_ = 705 nm and λ_pu2_ = 720 nm (or 725 nm depending on the tissue type) at a set λ_pr_ = 817 nm. The two pump-probe responses can be compared by incorporating the excitonic model^17^, which states that 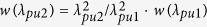. This mathematical relation describes the fact that under the Frenkel exciton model of chromophore broadening, the bandwidth of an exciton-broadened absorption line is directly proportional to its center frequency^17^. Therefore, the two pump-probe responses can be expressed as,


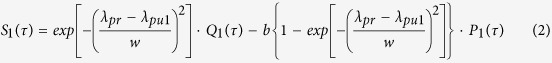



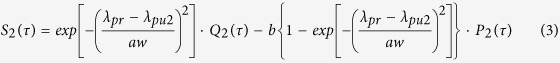


For simplicity, we substitute the notation of the ground state and excited state absorption endmembers with *Q* and *P*, respectively. This notation will be used below. We also allow *a* to be a free parameter in order to confirm the excitonic model, which should give 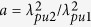. For our selection of pump wavelengths, we expect *a* = 1.06 (for λ_pu2_ = 720), or 1.04 (for λ_pu2_ = 725 nm).

**Figure 2:**
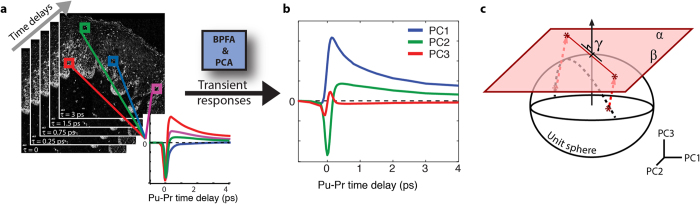
Data acquisition process and schematic representation of Gnomonic projections. **(a**) A stack of 55 images is collected (or 110 if the data from the two pump wavelengths is concatenated), with each image containing a different pump-probe time-delay (−1.5 to 4 ps). Inset shows the dynamics of four pixels in the composite stack. **(b)** Melanin dynamics (i.e., transient responses) measured from hundreds of thousands spatial pixels are process with a Beta Process Factor Analysis (BPFA) algorithm followed by Principal Component Analysis (PCA) to compute the Principal Components (PCs). **(c)** Projection of the data onto 3 PCs can be described in spherical coordinates. After normalization, points lay in the unit sphere which are then projected onto a tangent plane at a given point γ on the sphere, such that great circles (shortest paths) on a sphere correspond to straight lines in the new tangent plane with directions 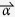 and 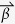.

The key outworking of this model is that the mixing between the endmembers (ESA and GSB, or P and Q, respectively) is nonlinear as a function of *w*. In the experiments described below, each signal vector S_1_ and S_2_ is a vector of measurements at 55 different values of τ at the same spatial location (see Fig. 2(a) Now consider the combination of the two measurements by concatenating them into a single vector, S_c_ = [S_1_, S_2_]. In the limit of large *w*: S_c_ → [Q1, Q2]; likewise, in the limit of small *w*: S_c_ → [P1, P2]. However, no value of ω can satisfy S_c_ = [(Q1 + P1)/2, (Q2 + P2)/2]. In other words, the concatenated measurement S_c_(*w*) cannot be expressed as a linear combination of the endmembers. Therefore, the path traced by S_c_ as a function of *w* is nonlinear, i.e. curved. The mathematical description provided below is designed to exploit this feature in order to confirm the model.

### Pump-probe signals’ mathematical representation and visualization

The pump-probe data collected for a given sample is a three-dimensional data cube, where two of the dimensions are the spatial (x and y) coordinates of the thin tissue biopsy specimen, and the third dimension is the pump-probe time delay, τ, which captures the molecules’ excited state dynamics (see Fig. 2(a)). To quantify the signals, we first seek to identify a dictionary of a small number of vectors (i.e., endmembers), Φ = [ϕ_1_ … ϕ_D_] *∈* 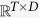, such that the signal vector for each pixel can be approximated by a nonnegative linear combination of endmembers. Here T is the number of time delays (55, or 110 if the data from the two pump wavelengths is concatenated, see methods section), and D is the number of identified endmembers. In other words, the pump-probe signal vector at one spatial pixel, *s*_*i*_ *∈* 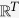, can be expressed as *s*_*i*_ = Φ*ν*_*i*_ + *ε*_*i*_ where *ν*_*i*_ *∈* 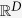 are the mixing coefficients of the endmembers/dictionary elements, and ε_i_ *∈* 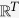 is noise/approximation error. In matrix notation this can be expressed as,


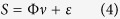


which defines the joint dictionary learning problem of finding Φ and *ν*, given a set of measurements, S. Here S = [s_1_, s_2_ ... s_N_] *∈* 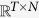, where N is the number of spatial pixels (e.g., 512 × 512 if we consider all pixels in one image), *ν* = [*ν*_1_, *ν*_2_ ... *ν*_N_] *∈* 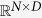, and ε = [ε_1_, ε_2_ ... ε_N_] *∈* 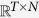. Clearly, our aim is to describe the data as fully as possible using this model; that is, to minimize the energy of ε.

The problem may be equivalently viewed as one of robust independent component analysis with nonnegativity constraints, which makes clear the link with principal component analysis (PCA), previously explored by Robles *et al*.^10^. Here we apply a Beta Process Factor Analysis (BPFA) algorithm^21^, which is specifically designed for joint robust nonegatively-constrained independent component analysis. Most notably, a nonnegative Beta Process prior is placed on the mixing coefficients *ν*, which, as well as enforcing nonnegativity, explicitly models the possibility that a given endmember may be entirely absent for a given spatial pixel.

After the independent components are determined for a given data set (e.g., cutaneous melanomas’ pump-probe signals with the pump set to 720 nm), the independent components are decomposed into an orthogonal basis set using PCA, with the output truncated to the third principal component (PC) (see Fig 2(b)). In all cases explored here, the top three PCs captured ≥97.5% of the variance. (Similar results can be obtained if PCA is performed directly on the data sets, which was the approach previously taken^10^). This procedure allows visualization of the data by projecting the resulting coefficients (projections of the data onto the 3 PCs) onto a unit sphere^10^. The resulting distribution is designed to preserve distances which is important for quantifying the relative concentrations of the endmembers; however, angles can be severely distorted. This is important: transition paths (i.e., mixtures) between two endmembers should be linear, but this corresponds to great circles on a sphere depending on the specific path (see Fig. 2(c)). Mixtures between multiple endmembers, which should produce changes in angle of the mixing paths, are difficult to identify using this spherical coordinate representation. It is worth noting that the same drawback is shared by some other approaches to endmember visualization, such as phasor analysis^24^.

Instead, we propose a different way of visualizing normalized rank 3 projections: the gnomonic projection^22^. In this representation, points are projected onto a tangent plane (defined by α and β in Fig. 2(c)) at a given point γ on the sphere, which we are free to choose (Fig. 2(c)). See supplemental material for more details. The primary appeal of the gnomonic projection is that great circles (shortest paths) on a sphere correspond to straight lines in the projection; in other words, this process preserves linearity through the transition paths or mixtures of two (or more) endmembers, given they possess linear mixing dynamics. This feature is paramount to confirm the nonlinear mixing paths predicted by our model. The gnomonic projections also facilitate visual identification of the endmembers (but do not directly compute them). Two downsides to the gnomonic projection are that only a hemisphere can be projected, and distance can be severely distorted. It should be noted, however, that projecting a sphere onto a plane always involves some kind of trade-off.

### Gnomonic projections of melanins’ excited state dynamics: cutaneous melanoma lesions

**Figure 3:**
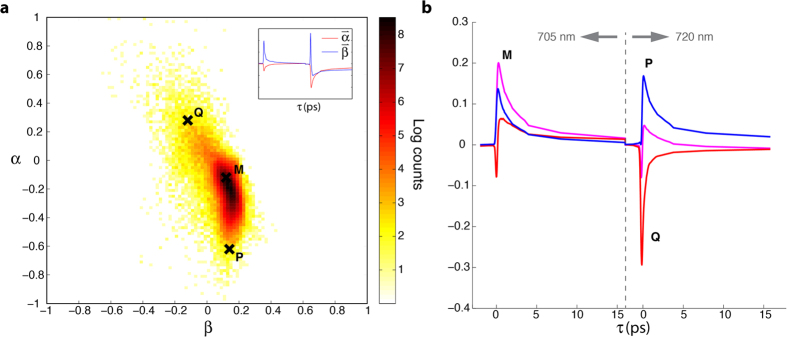
Gnomonic projection of the concatenated data (705/720 nm pump) from the cutaneous melanoma sample set. (**a)** Gnomonic projection of concatenated 705/720 nm pump, 817 nm probe data from cutaneous samples. Inset: Mutually orthogonal vectors 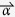 and 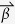. (**b)** Pump-probe response curves form the selected points.

First we consider a set of cutaneous melanoma samples, using the concatenated data from both pump wavelengths. As described in the methods section, these data consist of combining the pump-probe time traces acquired with a pump set to 705 nm and 720 nm from the same corresponding spatial points in the samples. The BPFA algorithm identifies 5 independent components, which are then decomposed into their principal components. The top 3 PCs capture 98% of the variance. The point of intersection for the gnomonic projection (γ) was chosen manually using 3D visualization tools. A histogram of the projection onto mutually orthogonal directions α and β in the gnomonic plane is displayed in Fig. 3(a). The inset shows the projected spectra along the α and β axis. The total number of pixels sampled is 154764, and frequencies in the histogram are displayed using a log-scale.

The results show a single transition path with a significant change in direction, suggesting no more than three potential endmembers. We note that the BPFA algorithm did not directly pinpoint the endmembers, thus these are approximated based on visual inspection. The points P, Q, and M in Fig. 3 were manually selected as these roughly delineate the ends of the distribution and the inflection point in the transition path, respectively. Keeping with the notation above (see Eqs 2 and 3), P = [P1, P2] and Q = [Q1, Q2]. Figure 3(b) shows the corresponding experimental pump-probe signals that map to these points in the gnomonic projection, which show a morphing of the transient dynamics from positive to predominantly negative along the transition path from P to Q for both wavelengths.

There are two possibilities for the observed change in direction of the distribution. First, this could result from the presence of three endmembers, where mixing is only possible between Q-M, and M-P, but not between Q-P. This would be very unlikely. Alternatively, the curved path of the distribution could results from differences in the rate of progress along the individual transition paths for the two pump wavelengths data sets. Physically, this may correspond to differences in the pump-selected underlying chromophores’ absorption bandwidth with respect to the pump and probe wavelength separation, as described in Eqs 1, 2, 3.

**Figure 4:**
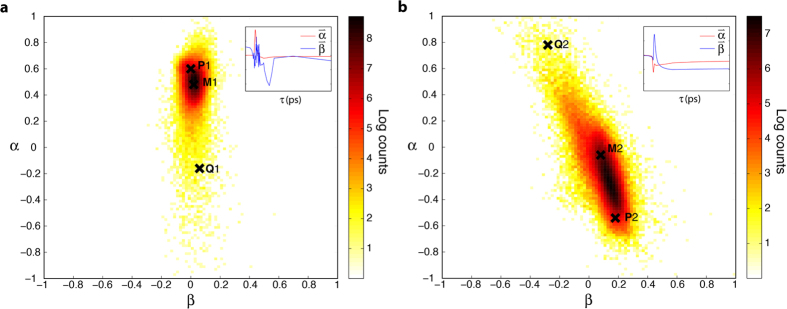
Gnomonic projection of the individual pump-probe wavelength combinations from the cutaneous melanoma sample set. Gnomonic projection of the (**a**) 705 nm and (**b**) 720 nm pump-probe data from cutaneous samples.

To test this hypothesis we repeat the same procedure as above, but now treating the 720 nm and 705 nm pump data separately. The results are shown in Fig. 4. Note that the noise-like appearance along 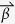 for the 705 nm data indicates that the signal energy is largely restricted to a single direction. The results show that both wavelengths follow a linear transition path, with very small, if any, change in direction, suggesting only two endmembers. The gnomonic plots also show the same reference points P, Q and M projected onto the new space. Note that P1 on Fig. 4(a), for example, only corresponds to the 705 nm portion of P in Fig. 3(b), etc. Points P1, P2, Q1 and Q2, once again, roughly point towards the ends of the distributions. However, the location of M differs significantly between the two plots (e.g., M2 is approximately midway between Q2 and P2 in Fig. 4(b), but M1 is much closer to P1 than Q1 in Fig. 4(a)). As we have previously discussed, the gnomonic projections are designed to preserve directionality but not distances, thus simple visual inspection can be misleading. Nevertheless, M can be represented as a combination of P and Q, as described by Eqs 2, 3, which should reveal if the mixing paths occur at different rates. Indeed, we find that the signal corresponding to M is given by M1_705_ ≈ 0.7P1 + 0.3Q1 and M2_720_ ≈ 0.5P2 + 0.5Q2, confirming that the curved path of the concatenated data (Fig. 3(a)) results from differences in the rate of progress along the individual transition paths.

### Gnomonic projections of melanins’ excited state dynamics: cutaneous blue nevi lesions

To further demonstrate that this behavior is consistent in melanins from other types of melanocytic lesions, we analyze cutaneous blue nevi lesions, acquired with pump wavelengths of 705 nm and 725 nm (see methods section). These lesions tend to be highly pigmented and exhibit remarkable pigment heterogeneity^25^.

**Figure 5:**
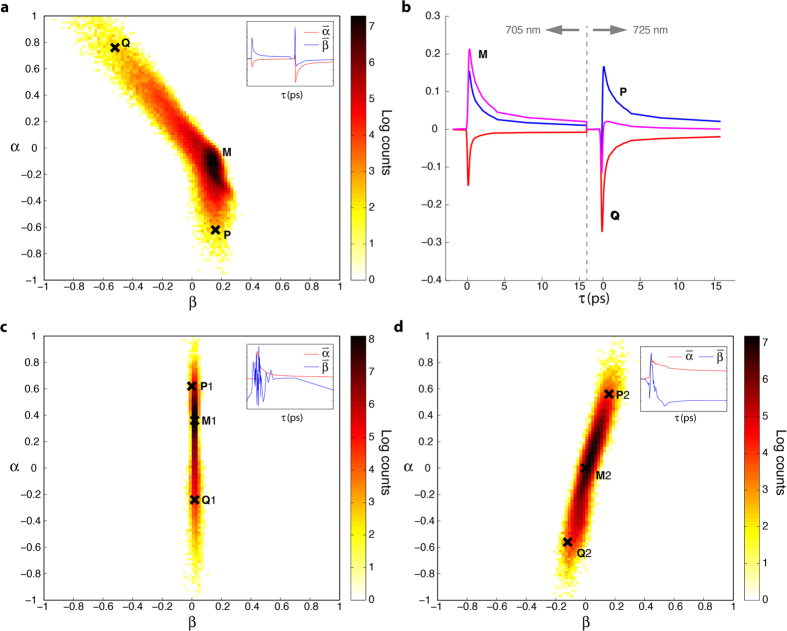
Gnomonic projections of cutaneous blue nevi sample set. (**a)** Gnomonic projection of concatenated 705/725 nm pump-probe data from blue nevi samples. (**b)** Pump-probe time delays from the selected points. Gnomonic projection of (**a**) 705 nm and (**b**) 725 nm pump-probe data.

Figure 5 shows the gnomonic projections of the concatenated 705 nm and 725 nm data. The same general trends are observed here, with slightly improved signal to noise ratio, which facilitates visualization of the inflection point, M. Again, no mixing occurs between points Q and P. Figure 5(b) shows the corresponding experimental pump-probe signals that map to points Q, M and P. The gnomonic projections of the individual data sets, shown in Fig. 5(c,d), once again show that the individual distributions are composed of a single, non-curved path. Finally, we calculate the mixing coefficients for the point M, which yields M1_705_ ≈ 0.8P1 + 0.2Q1 and M2_725_ ≈ 0.5P2 + 0.5Q2, once again providing clear evidence of a difference in the relative rate of mixing along the two paths.

### Modeling of the dual-wavelength transition path

Returning to Eqs 1, 2, 3, the data presented above can be used to directly extract the endmembers, P = [P1, P2] and Q = [Q1, Q2], leaving only *a* and *b* as unknowns. In our model, the spectral width of the underlying chromophores, *w*, is the only parameter that changes from one melanosome to the next. For example, changes in the extent of DHI(CA)/benzothiazine polymerization and metal ion content alter *w*^16^. The other parameters, *a* and *b*, are assumed to remain constant and can be estimated based on the specific transition paths (i.e., mixing rates of the endmembers). The assumption that these parameters are constant for different types of melanins is consistent with our working physical model since *a* should correspond to the squared ratio of the two pump wavelengths, and *b* is a relative weighting factor between the two endmembers that should not vary if the data is appropriately normalized (see methods).

To estimate *a* and *b*, first we take the discrete values of the parameter α (a row of the gnomonic plot), and estimate the position of the transition path as β^*^(α) = μ_α_, where μ_α_ is the mean of the β values at each α. We then vary the parameters (*a*, *b*) on a fine mesh (see supplemental material and Fig. S1), and for each mesh point compute the predicted transition path β^(*a*,*b*)^(α). Optimal values of *a* and *b* are obtained to an accuracy of 10^−2^ by minimizing the least squares error
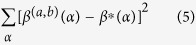


**Figure 6:**
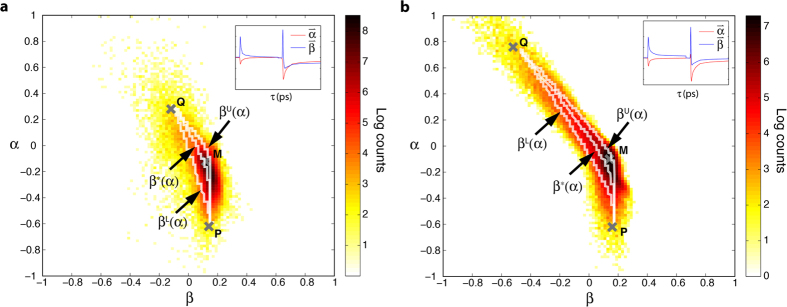
Modeled mixing paths. Optimally fitted mixing path to the (**a**) cutaneous samples and (**b**) blue nevi samples using the concatenated data.

The least squares process yields *a* = 1.32 and *b* = 0.26 for the cutaneous melanoma samples, and *a* = 1.28 and *b* = 0.37 for the blue nevi samples. The resulting mixing paths for both sample sets are illustrated in Fig. 6, which show good agreement with the data. Particularly note that the best-fit parameters indeed capture the curvature of the transition/mixing paths, in support of the model put forth here. To give confidence bounds, we also calculate β^L^(α) = μ_α_ − σ_α_ and β^U^ (α) = μ_α_ + σ_α_ (lower and upper bounds, respectively) where σ_α_ is the standard deviation of the β values at each α. In this case, however, we observed that the path trajectory does not greatly depend on parameter *b* (this parameter mainly affects the rate of progress along that path); thus we estimate the bounds (and hence error of parameter *a*), by assuming a constant value of *b* (set to the optimized value as determined above). See supplemental material for error surface plots, which show that *b* does not greatly affect the least square error of the upper and lower bounds. Estimates of the upper and lower confidence bounds for the cutaneous melanoma sample set yields *a* = [0.90, 2.10], and for the blue nevi *a* = [0.99, 1.80]. The resulting paths for the confidence bounds are also plotted in Fig. 6. Of significance, we note that the theoretical values of 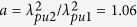 (for λ_pu2_ = 720), or 1.04 (for λ_pu2_ = 725 nm) are well within the bounds of our estimate, and further supports the excitonic model. Deviations from the expected central path could be due to contributions from exciting overlapping absorbers that have different central wavelengths; however, the higher mean value of *a* could also signify that other effects, such as the chemical heterogeneity (i.e., ‘chemical disorder’ model), are influencing the ultrafast pump-probe dynamic behavior.

## Discussion

In this work we have introduced a mathematical framework for the transient excited-state and ground-state photodynamics of melanin, measured via near-infrared pump-probe microscopy. The model depends on two endmembers, dominated by ground state bleaching and excited state absorption, and on the bandwidth of melanins’ underlying chromophores relative to the pump and probe wavelength separation. By combining pump-probe data acquired with different pump wavelengths, we were able to test and confirm specific predictions of the model; specifically, its nonlinear behavior with respect to *w*. The precise nonlinear mixing path of the endmembers also supports a recently developed excitonic model, which provides a well-defined link between the spectral properties of melanin’s underlying chromophores. Specifically, the model provides a mathematical (and thus quantitative) relationship describing the amount of chromophore broadening with respect to wavelength by invoking Frenkel excitons, which are characteristic of molecular aggregates^17^. The excitonic (or packing disorder) model is based on the interaction between geometric order and disorder of the pigment’s aggregate structure, and it has shown some promise in describing melanin’s broadband and monotonically decreasing optical absorption properties. The outworking of this particular model applied to the experiments here predicts a mixing path curvature that falls within the error bounds of our measurements (Fig. 6). The results presented here are significant as they lend further support to the idea that ‘chemical disorder’^26^,^27^,^28^ alone is inadequate to explain the optical properties of melanin; rather, packing disorder and excitonic couplings must be considered^17^.

It is important to compare the features of pump-probe microscopy with those of conventional broadband transient spectroscopy, and discuss why our approach is advantageous for this study. In general, there is a fundamental tradeoff between the two methods: broadband transient spectroscopy measures the pump-probe response over a large number of probe wavelengths and typically over a large spatial region (thus sampling an ensemble of molecules), at slow repetition rates (~1 kHz). Pump-probe microscopy, on the other hand, measures the response at a few wavelengths with high (<1 μm) spatial resolution, at much higher repetition rates (80 MHz). The features of pump-probe microscopy are important for medical imaging because they enable relatively fast sample throughput and visualization of subcellular structures for pathological evaluation. For this present study, these features allow us to sample hundreds of thousands of melanosomes naturally present in humans, leading to the assessment of the nonlinear mixing paths. With conventional broadband transient spectroscopy, this information would be extremely cumbersome to sample, and the nonlinear mixing paths would likely not be resolved as the features would be averaged out over the ensemble measurement^29^. Ultimately, pump-probe microscopy enables us to (1) see the excitonic behavior and (2) see that the behavior persists across a wide range of naturally formed melanins.

Another important factor enabling this study is the use of the gnomonic projections to analyze the data. This approach transforms great circles on a sphere to straight lines, facilitating identification of a distribution’s endmembers and their mixing paths. Moreover, the gnomonic approach is not restricted to visualization of 3D data, and can thus be extended to project a half-hypersphere of any dimension onto a hyperplane. For example, a 4D hypersphere would project into 3D space such that transition paths on the 4D hypersphere become straight lines in 3D space. Thus, this approach can be a useful tool for detecting linear paths of normalized mixing coefficients of any dimension size. Moreover, this approach has implications beyond melanin analysis. Principal component analysis is widely and successfully used in various disciplines of analytical chemistry, for example spectroscopy and chromatography^30^. A main goal of analytical chemistry, with notable applications in forensics^31^, is the determination of the chemical species of a sample. This task commonly requires the determination of the proportions of the constituent components of the material, which makes it sensible, and even vital, to normalize the data. Normalization projects the data onto a hypersphere, and gnomonic projection then provides a natural way to visualize the normalized data in a way which preserves angle information. This property makes gnomonic projection an attractive generic tool for mixing path analysis of heterogeneous chemicals and multidimensional endmember identification.

One of the original objectives of the study was to determine if combination of multiple pump-wavelengths could reveal more information regarding different types of melanins (i.e., could the use of two pump-wavelengths reveal more than two endmembers?). However, despite giving the endmember set every opportunity to expand to higher dimensionality (using the BPFA algorithm), there is no evidence of anything other than a single transition/mixing path. Therefore, while the use of multiple pump-wavelengths was paramount to validate our model of pump-probe signals (and the excitonic model), it is unlikely that the added complexity and time required to conduct the experiments would be of significant value to pump-probe microscopy imaging of pigmented lesions for diagnostic purposes. Nevertheless, this could be a useful tool from a materials/chemical analysis perspective.

An interesting note regarding these data concerns the potential for the two endmembers of a single pump-probe wavelength combination to completely cancel each other. As described in the methods section, here we required each spatial pixel’s total signal energy to be above a certain threshold for the 705 nm pump data *or* the 720 nm pump data for inclusion in the histograms in Figs 3,4,5 and 6. Since the mixing paths for the two pump-wavelengths occurs at different rates, thresholding in this manner significantly reduces the likelihood of signal cancelation; but again, this cannot always be avoided using a single pump-probe wavelength combination. This is particularly detrimental for the 705 nm pump data. Supplemental Fig. S2 and S2 clearly show that complete sections of the 705 nm histogram are lost when analyzing these data without taking the 720 nm data into account. Thresholding the 720 nm data without taking the 705 nm data into account, on the other hand, does not significantly alter the overall interpretation of the pigment distribution (though some caution is still warranted when analyzing these signals alone, depending on the particular dynamics of the endmembers, see Fig. S3). This, again, points towards the 720 nm pump being a better choice for pump-probe imaging of pigmented lesions.

In conclusion we have shown that pump-probe dynamics of melanin result from differences in the spectral bandwidths of the underlying ensemble of chromophores, as well as the spectral proximity of the pump and probe. The mathematical framework used here models the individual chromophores as Gaussian absorbers with their bandwidth related by the Frenkel exciton model. The results further support the packing disorder model of melanin absorption, though the deviations from the exact predicted path are likely indicative of effects of chemical heterogeneity (i.e., ‘chemical disorder’ model).

## Methods

### Experimental setup

The pump-probe set up has been described in detail elsewhere^6^,^8^. In short, the output of a mode-locked Ti:Sapphire laser, tuned to 817 nm (bandwidth Δλ ~ 7 nm), is split into two beams: One is used to pump an optical parametric oscillator which is tuned to the desired pump wavelength (705, 720 or 725 nm, Δλ ~ 5 nm). The output is then modulated at 2 MHz using an acousto-optic modulator. The second beam from the Ti:Sapph (probe beam) is sent to a delay-line to control the time of arrival of the probe with respect to the pump. The pump and probe beams are recombined and sent to a custom-built scanning microscope. We use a 20X objective (0.8NA), and the pulses are ~140 fs each at the sample. When a nonlinear interaction between the two beams occurs in the sample, the pump modulation is transferred to the probe beam, which is then detected using a lock-in-amplifier. The sample is raster scanned at each desired pump-probe time delay which yields a three dimensional data set: two dimensions are the spatial (x-y) dimension of the sample (512 × 512 pixels per image) and the third is the pump-probe time delay (T = 55) (See Fig 2(a)). The pump and probe powers at the sample are kept below 0.3 mW (peak intensity ~2.5 GW/cm^2^), each, to avoid damaging the melanins^7^.

### Specimens

Images were acquired from thin (5 μm) sections of unstained tissue, from archived pathology specimens provided under an IRB-approved protocol. The specimen and wavelengths selected are: (1) N = 9 samples of pigmented cutaneous melanoma lesions at both 705 nm and 720 nm pump wavelengths, and (2) N = 4 samples of pigmented cutaneous blue nevi lesions at 705 nm and 725 nm. Data from the two tissue types were kept separate.

### Data processing

The following preprocessing steps were carried out (in the order given) for each image for all pump-probe wavelength combinations. *Thermal offset correction:* The recorded pump-probe signals are a superposition of ultrafast transient behavior and a long-lived thermal offset. For a given image and wavelength, an approximation of the thermal offset (assumed variable across the image) is obtained using a rank 6 PCA projection of the signal at negative time delay (i.e., probe precedes the pump). This approximation of the thermal offset is then subtracted from each time delay slice. *Smoothing*: To help suppress noise and reduce potential effects form image miss-registration, the datacube for each image/wavelength is smoothed using a 3D Gaussian filter of size 3 × 3 × 3. *Ink removal*: As is standard practice for skin biopsy, many of the specimens had been marked with surgical ink. Surgical ink typically also has a significant absorptive response which would interfere with that of melanin. Regions of ink are located by visual inspection, and then a mask is applied to discard image regions with obvious surgical ink contributions.

The following procedure is taken to analyze the combined data from all images in a given study, both for each wavelength separately, and when the wavelengths are combined. The wavelengths are combined by concatenating the signal vectors of each wavelength for a given pixel. Concatenating in this way for all pixels, we obtain a datacube of size 512 × 512 × 55 W, where W is the number of wavelengths (W = 2 in our case). Misalignment of images from different wavelengths is corrected either using autocorrelation-based registration, or by visual inspection. Since the samples are static, this is sufficient to ensure there are no issues with image alignment or co-registration with the two pump-probe wavelength measurements of a given sample.

To address the potential lack of standardization of signal amplitudes across images, the mean noise level of each image is estimated from the image intensity at negative time delay. This estimate is then used as a normalization, so that signal amplitudes represent SNR on a standardized scale.

Following Robles *et al*.^24^, high SNR pixels are selected by thresholding according to the Euclidean norm of the signal vector for each pixel. A threshold of μ + 2σ is used, where μ and σ are the mean and standard deviation of the pixel energies respectively. Thresholding is based on either of the two pump-probe time delays at a given spatial pixel passing this threshold; that is, either the 705 nm pump data *or* the 720 nm pump data have to satisfy this criterion at a given spatial pixel, but not both.

## Additional Information

**How to cite this article**: Thompson, A. *et al*. Dual-wavelength pump-probe microscopy analysis of melanin composition. *Sci. Rep.* **6**, 36871; doi: 10.1038/srep36871 (2016).

**Publisher’s note**: Springer Nature remains neutral with regard to jurisdictional claims in published maps and institutional affiliations.

## Supplementary Material

Supplementary Information

## References

[b1] D’Ischia M. . Melanins and melanogenesis: from pigment cells to human health and technological applications. Pigment Cell Melanoma Res 28, 520–544 (2015). 26176788 10.1111/pcmr.12393

[b2] Simon J. D. & Peles D. N. The red and the black. Accounts of Chemical Research 43, 1–5 (2009). 10.1021/ar100079y 20734991

[b3] Plonka P. M. . What are melanocytes really doing all day long? Experimental Dermatology 18, 799–819 (2009). 19659579 10.1111/j.1600-0625.2009.00912.x PMC2792575

[b4] D’Ischia M. . Melanins and melanogenesis: methods, standards, protocols. Pigment Cell Melanoma Res 26, 616–633 (2013). 23710556 10.1111/pcmr.12121

[b5] Fischer M. C., Wilson J. W., Robles F. E. & Warren W. S. Invited Review Article: Pump-probe microscopy. Rev Sci Instrum 87, 031101 (2016). 27036751 10.1063/1.4943211 PMC4798998

[b6] Fu D., Ye T., Matthews T. E., Yurtsever G. & Warren W. S. Two-color, two-photon, and excited-state absorption microscopy. J Biomed Opt 12, 054004 (2007). 17994892 10.1117/1.2780173

[b7] Simpson M. J. . Near-Infrared Excited State Dynamics of Melanins: The Effects of Iron Content, Photo-Damage, Chemical Oxidation, and Aggregate Size. J. Phys. Chem. A 118, 993–1003 (2014). 24446774 10.1021/jp4107475 PMC3983346

[b8] Matthews T. E., Piletic I. R., Selim M. A., Simpson M. J. & Warren W. S. Pump-probe imaging differentiates melanoma from melanocytic nevi. Science Translational Medicine 3, 71ra15–71ra15 (2011). 10.1126/scitranslmed.3001604 PMC3371363 21346168

[b9] Wilson J. W. . Imaging microscopic pigment chemistry in conjunctival melanocytic lesions using pump-probe laser microscopy. Invest Ophthalmol Vis Sci 54, 6867–6876 (2013). 24065811 10.1167/iovs.13-12432 PMC3805089

[b10] Robles F. E. . Pump-probe imaging of pigmented cutaneous melanoma primary lesions gives insight into metastatic potential. Biomedical Optics Express 6, 3631–3645 (2015). 26417529 10.1364/BOE.6.003631 PMC4574685

[b11] Robles F. E., Wilson J. W. & Warren W. S. Quantifying melanin spatial distribution using pump-probe microscopy and a 2-D morphological autocorrelation transformation for melanoma diagnosis. J Biomed Opt 18, 120502 (2013). 24296994 10.1117/1.JBO.18.12.120502 PMC3843113

[b12] Matthews T. E. . *In vivo* and *ex vivo* epi-mode pump-probe imaging of melanin and microvasculature. Biomedical Optics Express 2, 1576–1583 (2011). 21698020 10.1364/BOE.2.001576 PMC3114225

[b13] Wilson J. W. . Comparing *in vivo* pump–probe and multiphoton fluorescence microscopy of melanoma and pigmented lesions. J Biomed Opt 20, 051012 (2015). 25415567 10.1117/1.JBO.20.5.051012 PMC4409034

[b14] Piletic I. R., Matthews T. E. & Warren W. S. Probing near-infrared photorelaxation pathways in eumelanins and pheomelanins. J. Phys. Chem. A 114, 11483–11491 (2010). 20882951 10.1021/jp103608d PMC3334281

[b15] Simpson M. J. . Pump–Probe Microscopic Imaging of Jurassic-Aged Eumelanin. J. Phys. Chem. Lett. 4, 1924–1927 (2013). 23847720 10.1021/jz4008036 PMC3704187

[b16] Meng S. & Kaxiras E. Theoretical models of eumelanin protomolecules and their optical properties. Biophysical Journal 94, 2095–2105 (2008). 17993493 10.1529/biophysj.107.121087 PMC2257886

[b17] Chen C.-T. . Excitonic effects from geometric order and disorder explain broadband optical absorption in eumelanin. Nature Communications 5, 1–10 (2014). 10.1038/ncomms4859 24848640

[b18] Fidder H., Knoester J. & Wiersma D. A. Optical properties of disordered molecular aggregates: a numerical study. The Journal of chemical physics 9, 7880–7890 (1991).

[b19] Markovitsi D., Gallos L. K., Lemaistre J. P. & Argyrakis P. Degeneracy, orientational disorder and chromophore size effects on Frenkel excitons in columnar mesophases. Chemical Physics 269, 147–158 (2001).

[b20] Spano F. C. The spectral signatures of Frenkel polarons in H-and J-aggregates. Accounts of Chemical Research 43, 429–439 (2009). 10.1021/ar900233v 20014774

[b21] Xing Z., Zhou M., Castrodad A., Sapiro G. & Carin L. Dictionary Learning for Noisy and Incomplete Hyperspectral Images. SIAM Journal on Imaging Sciences 5, 33–56 (2012).

[b22] Snyder J. Map projections - a working manual . (United States Government Printing Office, 1987).

[b23] Prampolini G., Cacelli I. & Ferretti A. Intermolecular interactions in eumelanins: a computational bottom-up approach. I. small building blocks. RSC Adv. 5, 38513–38526 (2015).

[b24] Robles F. E., Wilson J. W., Fischer M. C. & Warren W. S. Phasor analysis for nonlinear pump-probe microscopy. Opt. Express 20, 17082–17092 (2012).

[b25] González-Cámpora R., Galera-Davidson H., Vázquez-Ramírez F. J. & Díaz-Cano S. Blue nevus: classical types and new related entities. A differential diagnostic review. Pathol. Res. Pract. 190, 627–635 (1994). 7984522 10.1016/S0344-0338(11)80402-4

[b26] Tran M. L., Powell Ben, J. & Meredith P. Chemical and Structural Disorder in Eumelanins: A Possible Explanation for Broadband Absorbance. Biophysj 90, 743–752 (2006). 10.1529/biophysj.105.069096 PMC1367100 16284264

[b27] Meredith P. . Towards structure–property–function relationships for eumelanin. Soft Matter 2, 37–44 (2006). 10.1039/b511922g 32646091

[b28] D’Ischia M., Napolitano A., Pezzella A., Meredith P. & Sarna T. Chemical and Structural Diversity in Eumelanins: Unexplored Bio-Optoelectronic Materials. Angew. Chem. Int. Ed. 48, 3914–3921 (2009). 10.1002/anie.200803786 PMC2799031 19294706

[b29] Hartland G. V. Ultrafast studies of single semiconductor and metal nanostructures through transient absorption microscopy. Chemical Science 1, 303–309 (2010).

[b30] Malinowski E. R. Factor analysis in chemistry . (Wiley, 2002).

[b31] Blackledge R. D. Forensic analysis on the cutting edge: new methods for trace evidence analysis . (Wiley, 2007).

