# Supplementary material for: Dual-wavelength pump-probe microscopy analysis of melanin composition
Source: Sci Rep. 2016 Nov 11;6:36871. doi: 10.1038/srep36871 (PMC5104978; doi:10.1038/srep36871)
Supplement: Supplementary Information [file srep36871-s1.pdf]

Supplemental Material: Dual-wavelength pump-probe microscopy analysis of melanin composition

Andrew Thompson, Francisco E. Robles, Jesse W. Wilson, Sanghamitra Deb, Robert Calderbank and Warren S. Warren*

*Corresponding author: warren.warren@duke.edu

Supplementary Information:

Surface error plots. Supplementary Fig. S1 shows the error as a function of varying the free parameters *a* and *b* using Eqs 2-3 to the mean path and to the upper and lower paths, as shown in Fig. S1. Specifically, the parameters *a* and *b* were optimized over an adaptively-chosen range, and the least squares error was evaluated on a 20x20 fine mesh of (*a,b*) points within the range. The ranges of the fine mesh for each particular determined path (i.e., mean, lower and upper bounds) are shown in Fig. S1.

The value of using two different wavelengths. One of the original goals of this study was to determine if using two sets of pump wavelengths provided evidence of more than two endmembers. However, despite giving the endmember set every opportunity to expand to higher dimensionality, there is no evidence of anything other than a single transition path. In fact, we have seen that interpreting directional information is actually harder in the case of concatenated data due to differences in progress along the transition paths for the two wavelengths.

Recall that the selection of pixels was based on an OR gate approach, where a pixel is 'let through' the gate if the SNR of either the 705 nm *or* 720/725 nm signal exceeds a certain threshold. In other words, the data analysis for separate wavelengths presented above relied upon the data from both wavelengths, specifically for the pixel selection. We explored the importance of combining SNR information in this way by projecting only those pixels that would be selected based on the data for that particular wavelength (using the same gnomonic plane). Figure S2 compares the two methods of projection for the cutaneous melanoma

sample set. For each of the two wavelengths, the left-hand plot uses the OR gate, the middle plot performs separate thresholding and the right-hand plot displays the pixels caught by the OR gate but missed by separate thresholding. Approximately 30% and 45% of the pixels obtained using the OR gate are missed when separately sampling 705nm and 720nm respectively. Corresponding plots for the blue nevi sample set are in Fig. S3. Approximately 33% and 44% of the pixels obtained using the OR gate are missed when separately sampling 705nm and 725nm respectively.

Most significantly, we notice that, in both studies, whole sections of the 705nm transition path are missed by separate thresholding. An inspection of Figs. 3(b) and 5(b) reveals why: opposite ends of the transition path are negative correlated (especially so for the blue nevi data), and so signals midway along the transition path would exhibit cancellation and therefore would be expected to have much smaller amplitudes relatively. For the cutaneous melanoma data set with the 720 nm pump, the OR gate seems to make less difference. It seems that the effect of cancellation may be mitigated by the fact that there is so much signal content precisely for mixing ratios where the amplitude is reduced. For the blue nevi data set with a 725 nm pump, if the OR gate is not used the distribution appears to accumulate in two distinct regions. Signal cancellation is almost certainly the explanation for the relatively low density of pixels midway along the transition path in the case of separate thresholding.

To further investigate this effect, we plot the average signal amplitude of the data across the gnomonic plane, for both studies, and with both separate and concatenated wavelengths (Figs. S4 and S5). Both figures illustrate clearly the cancellation effect for separate wavelengths, and also the advantage to be gained by using both wavelengths.

Details on the gnomonic projection. The gnomonic projection, with its enigmatic silent ‘g’, is said to be the oldest map projection, developed by Thales in the 6th century BC. Due to its property of projecting great circles to straight lines, it is used in navigation to find shortest routes. Other uses are in seismology (seismic waves tend to trace great circles), astronomy and photography (where it is known as rectilinear projection).

The projection is realized by projecting onto the tangent plane to the sphere at a given point $\gamma = (\gamma_1, \gamma_2, \gamma_3)$ (which can be chosen arbitrarily). A point $x = (x_1, x_2, x_3)$ on the sphere is projected by taking the straight line joining the center of the sphere (0, 0, 0) and x, and extrapolating it onto the tangent plane. The result is what one would see if one were standing at the center of the sphere looking outwards towards γ. The plane passing through γ from the origin splits the sphere into two hemispheres, and only the hemisphere which contains γ can be projected.

Given a choice of γ, the gnomonic projection $\wp$ is in fact very simple, being given by

$$\wp : x \to \frac{x}{\gamma^T x}$$

Two perpendicular unit vectors in the gnomonic plane are given by

$$\alpha = \frac{1}{\sqrt{\gamma_1^2 + \gamma_3^2}}(\gamma_3, 0, -\gamma_1), \quad \beta = \frac{1}{\sqrt{\gamma_1^2 + \gamma_3^2}}(\gamma_1\gamma_2, -\gamma_1^2 - \gamma_3^2, \gamma_2\gamma_3)$$

and projected points can then be plotted in the (α,β) plane.

It is easy to show that great circles are mapped to straight lines. A great circle is the intersection of a plane with the sphere, and so is defined by the equations

$$b^T x = 0, \quad \|x\|_2 = 1$$

where b is a vector normal to the plane, and $b \neq 0$. Meanwhile, the tangent plane at point γ has a normal vector that passes through the point from the origin, and so has equation $\gamma^T x = 1$. The gnomonic projection of the great circle follows a ray from the origin and so preserves the $b^T x = 0$ constraint, while replacing the $\|x\|_2$ constraint with $\gamma^T x = 1$. The gnomonic projection of the great circle is therefore defined by

$$b^T x = 0, \quad \gamma^T x = 1$$

which is the equation of a straight line in 3D space, provided $b \neq \gamma$.

Supplementary Figures:

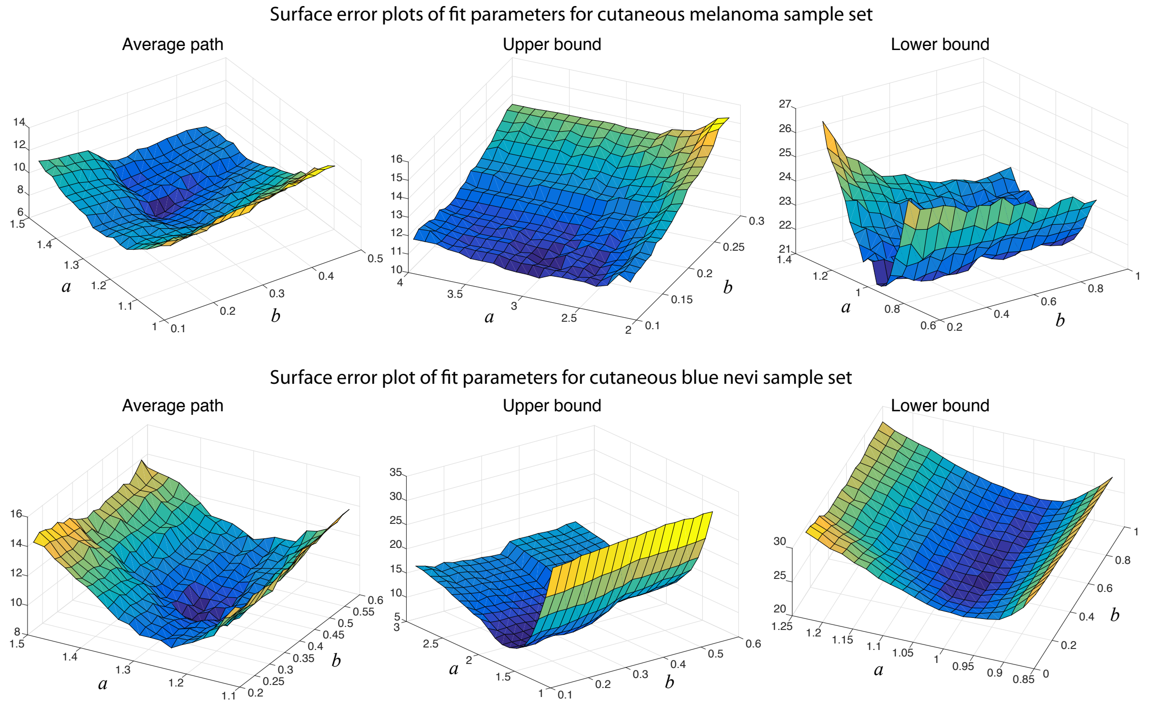

Supplementary Figure 1| Surface error plots of free parameters for the cutaneous melanoma data set (above) and cutaneous blue nevi (below).

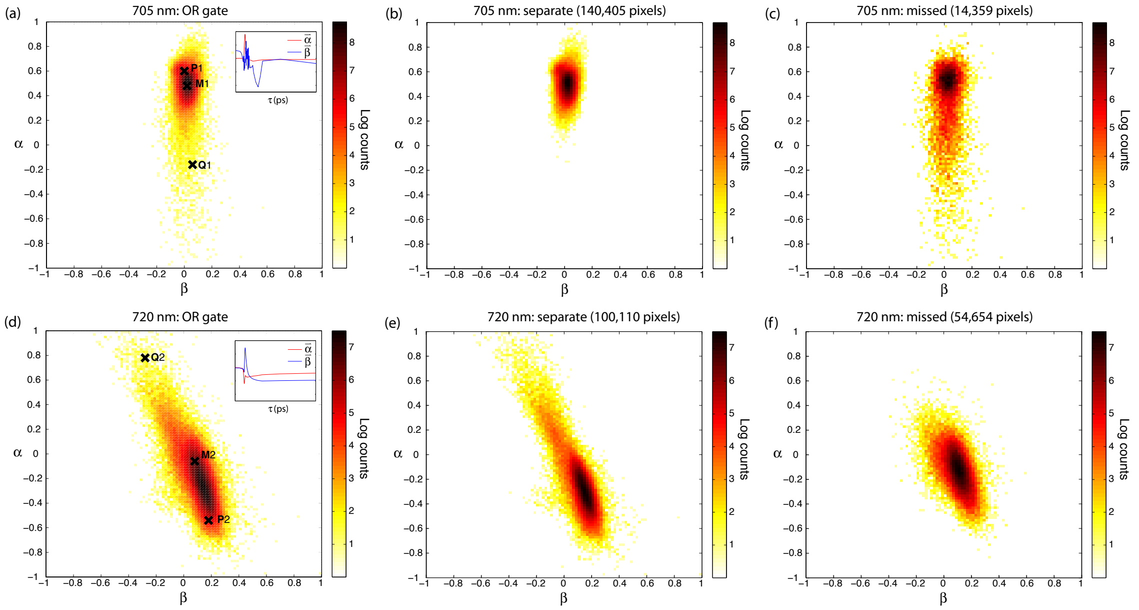

Supplementary Figure 2| OR gate versus separate thresholding for cutaneous melanoma sample set.

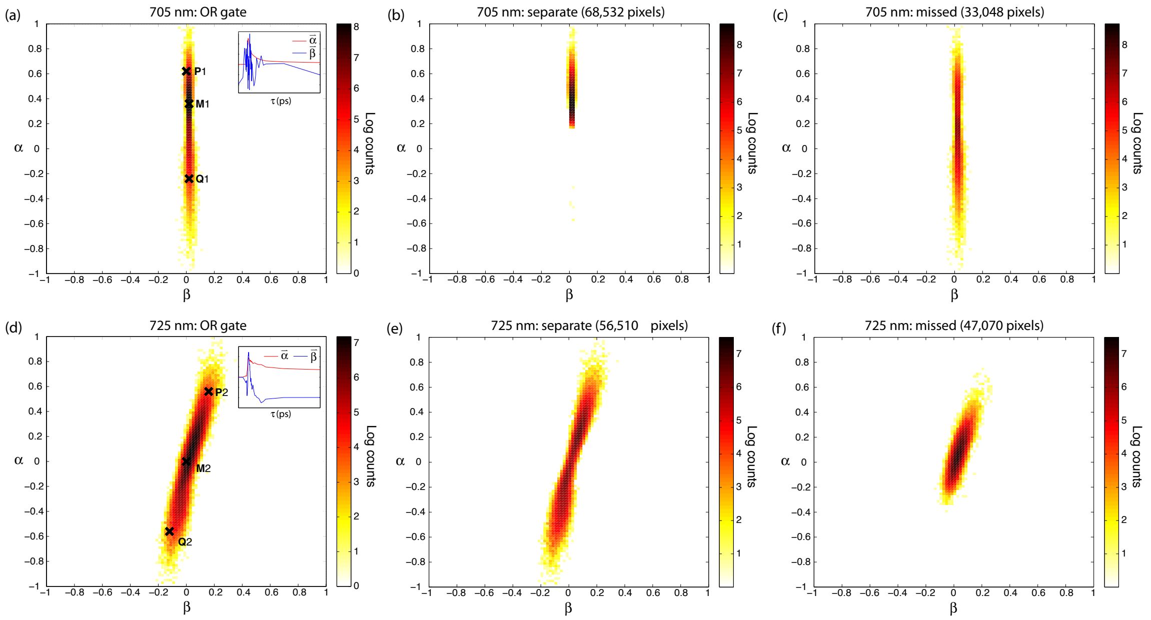

Supplementary Figure 3| OR gate versus separate thresholding for cutaneous blue nevi sample set.

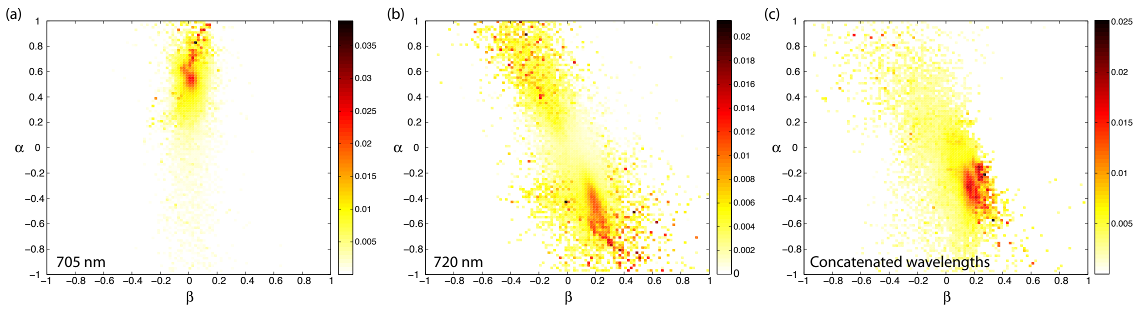

Supplementary Figure 4| Signal intensity across the gnomonic plane for the cutaneous melanoma sample set

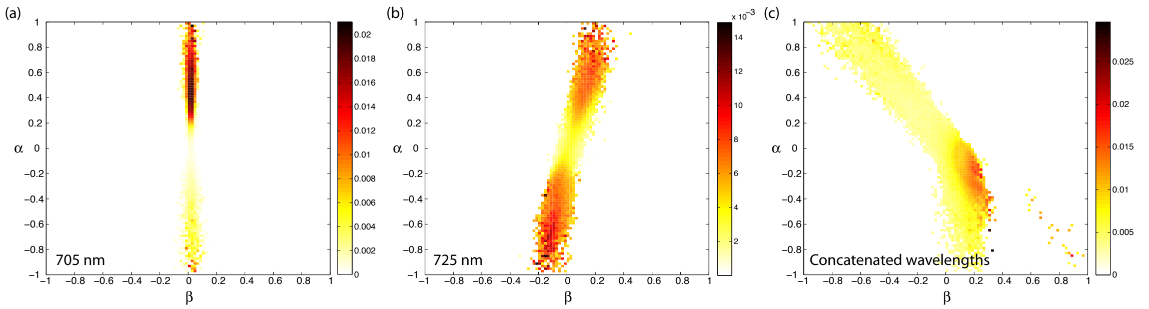

Supplementary Figure 5| Signal intensity across the gnomonic plane for the cutaneous blue nevi sample set
